# Are atypical lymphocytes a new predictive factor in the development of COVID-19?

**DOI:** 10.1590/0037-8682-0154-2022

**Published:** 2022-09-19

**Authors:** Claudio Bruno Silva de Oliveira, Joelma Maria de Araújo Andrade, Jonas Ivan Nobre Oliveira

**Affiliations:** 1 Hospital Pediátrico Maria Alice Fernandes, Natal, RN, Brasil.; 2 Hemocentro Dalton Cunha, Natal, RN, Brasil.; 3 Universidade Federal do Rio Grande do Norte, Departamento de Biofísica e Farmacologia, Natal, RN, Brasil.

**Dear Editor,**

We had the great opportunity to read the article titled “Bedside echocardiography to predict mortality of COVID-19 patients beyond clinical data: Data from the PROVAR-COVID study,” which was published in the *Journal of the Brazilian Society of Tropical Medicine* by Pimentel et al^1^. Publications such as this are of great importance as they present new tools for diagnosing and monitoring the clinical evolution of patients. The approach presented here is significant in the context of new diseases, especially when they are accompanied by effects similar to those observed in COVID-19^2^.

The etiological agent of this disease is SARS-CoV-2 and its variants, which are single-stranded positive RNA viruses with genomes that encode four major structural proteins: spike, membrane, envelope, and nucleocapsid proteins. Two other members of this viral family include Severe Acute Respiratory Syndrome-associated coronavirus (SARS-CoV) and Middle East Respiratory Syndrome-associated coronavirus (MERS-CoV), which share 79% and 50% of their genetic identity with SARS-CoV-2, respectively^3^. Viral transmission usually occurs horizontally from infected symptomatic or asymptomatic^4^ individuals.

In addition to the high number of reported cases, COVID-19 has killed approximately 6 million people worldwide. Initially, symptoms appeared in the form of severe pneumonia, especially in older adults^2^. However, this characteristic has changed with the emergence of new variants^5^ and advanced vaccination campaigns. Therefore, the disease currently presents with nonspecific symptoms, making it important to develop predictive tools such as those mentioned by Pimentel et al^1^.

Several groups have proposed alternatives aimed at establishing conditions that can be considered prognostic and risk factors in the evolution of patients with COVID-19, and highlighted hypertension, diabetes, and obesity^1^ ^,^ ^6^ ^,^ ^7^. Several alternatives appear among patient evolution markers, ranging from abnormalities in electrocardiograms^1^, oxygenation patterns, and renal, hepatic, vascular, and hemostatic function. In this context, inflammation, muscle, and cardiac injury biomarkers, in addition to damaged liver and kidney function and hemostatic activity, are very useful. Moreover, serum ferritin, interleukin (IL)-6, and IL-10 levels are strong predictors of severe disease^1^ ^,^ ^6^ ^,^ ^7^.

However, changes in clinical patterns caused by SARS-CoV-2 variants present new challenges for monitoring certain parameters, such as hematologic analysis^5^. We suggest that these changes should always be considered in order to appropriately update analysts to facilitate a quick response to the abnormalities observed in COVID-19.

Laboratory models are constantly being adapted to better understand current and emerging diseases. A variety of experimental models provide information for hematological analysis of leukocyte migration in various diseases^6^. In many situations, the observed pattern provides insight into the nature and pathology of acute or chronic viral infections.

Changes in lymphocyte morphology with the formation of pleomorphic cells known as atypical or reactive lymphocytes were initially poorly reported, and their clinical significance was underestimated at the beginning of the pandemic when most reported observations were essentially of lymphopenia^8^. This view has been maintained despite the fact that atypical lymphocytes are considered common as well as relevant to other viruses^9^.

Herein, we present recent data and review previously established concepts. Authors such as Pozdnyakova et al.^4^ conducted multivariate analyses and reported that the presence of atypical lymphocytes, particularly the plasmacytoid form, is a predictive factor for COVID-19. In addition, the authors noted that changes in lymphocyte patterns made it possible to determine the different stages of COVID-19. In these cases, the observation of atypical lymphocytes was more evident in the initial or mild stages of the disease. However, this feature was not observed during the most severe stages.

Similarly, Sugihara et al.^10^ demonstrated that atypical lymphocytes were frequently observed in patients with COVID-19. These authors pointed out that most patients included in the study had better clinical evolution when these atypical lymphocytes were present. The authors also pointed out that low numbers of CD8+ T lymphocytes were detected in patients with severe COVID-19^11^, suggesting a relationship between T cells and disease severity^10^, which is essential because a complex disease is expected to have a number of pathophysiological abnormalities.

Gelarden et al. (2021)^12^ also referred to the frequent observation of atypical lymphocytes in patients with COVID-19 and reported that this atypia is usually accompanied by lymphopenia. Interestingly, the authors also showed that atypical lymphocytes are very common in severe pneumonia caused by SARS-COV-2 in bronchoalveolar lavage samples. Furthermore, the authors clarified that the appearance of these lymphocytic forms in the bronchoalveolar lavage of a patient who was previously negative for the disease should stimulate a new collection to search for the virus. 

In our findings, we reiterate that the previously established parameters need to be verified in the hematologic analysis of patients with COVID-19. We suggest that mere observation of the presence of lymphopenia is insufficient and may lead to considerable misdiagnosis; therefore, an active search for the presence of atypical forms of lymphocytes is essential and may be fundamental to adequately monitor patients hospitalized with COVID-19.

This new virus will bring about new challenges and constant changes in laboratory standards. Therefore, we suggest that new investigations and analyses should be performed and presented on a regular basis to keep clinical analysts apprised, thereby facilitating an appropriate clinical diagnosis.
